# Dominant Attractor in Coupled Non-Identical Chaotic Systems

**DOI:** 10.3390/e24121807

**Published:** 2022-12-11

**Authors:** Dorsa Nezhad Hajian, Sriram Parthasarathy, Fatemeh Parastesh, Karthikeyan Rajagopal, Sajad Jafari

**Affiliations:** 1 Department of Biomedical Engineering, Amirkabir University of Technology (Tehran Polytechnic), Tehran 159163-4311, Iran; 2 Centre for Computational Modelling, Chennai Institute of Technology, Chennai 600069, Tamil Nadu, India; 3 Centre for Nonlinear Systems, Chennai Institute of Technology, Chennai 600069, Tamil Nadu, India; 4 Health Technology Research Institute, Amirkabir University of Technology (Tehran Polytechnic), Tehran 159163-4311, Iran

**Keywords:** non-identical chaotic oscillators, coupling, dominant attractor, largest Lyapunov exponent

## Abstract

The dynamical interplay of coupled non-identical chaotic oscillators gives rise to diverse scenarios. The incoherent dynamics of these oscillators lead to the structural impairment of attractors in phase space. This paper investigates the couplings of Lorenz–Rössler, Lorenz–HR, and Rössler–HR to identify the dominant attractor. By dominant attractor, we mean the attractor that is less changed by coupling. For comparison and similarity detection, a cost function based on the return map of the coupled systems is used. The possible effects of frequency and amplitude differences between the systems on the results are also examined. Finally, the inherent chaotic characteristic of systems is compared by computing the largest Lyapunov exponent. The results suggest that in each coupling case, the attractor with the greater largest Lyapunov exponent is dominant.

## 1. Introduction

Chaos theory is an inviting subject of study in nonlinear dynamics. The concepts in line with chaotic dynamics, such as strange attractors, fractal dimensions, Lyapunov exponents, and synchronization, have been extensively addressed in every research field [1,2]. For example, in neuroscience, the presence of deterministic chaos has been proved both experimentally and theoretically [3,4]. Networks consisting of chaotic oscillators have also received considerable attention over the past decades [5,6].

In the context of networks, coupling and its configuration are inseparably studied. Adjustment of coupling strength may result in a correlated in-time behavior, universally known as synchronization [7]. Such a collective state of coupled systems indicates dynamical interactions, having a critical role in many natural networks [8]. For example, it is vital in nervous system tasks such as the human memory process or enhancement of live verbal communication [9,10,11]. Regarding chaotic systems, two trajectories starting from initial conditions tend to diverge exponentially over time [12,13]. As a result, it was first realized that chaotic systems intrinsically defy synchronization until, in 1990, Pecora and Carroll [14] proved quite the contrary. They showed that synchronization is achievable by drive-response coupling of two identical chaotic systems [12].

Chaotic systems exhibit various forms of synchronization such as phase [15], antiphase [16], lag [17], explosive [18], cluster [19,20], generalized [21], and complete [22]. Complete synchronization, the perfect hooking of chaotic trajectories, is only achievable in identical systems [13,23]. In such systems, attractors structures begin to assimilate until they match completely [24]. However, when oscillators are non-identical, even the classic definition of the synchronization manifold is not analytically valid [23]. Consequently, few studies have discussed this collective behavior in non-identical chaotic systems [25,26,27]. Some studies have reported various indexes for synchronization detection [28,29]. Non-identical systems have also been studied from views other than synchronization and its related concepts. Most of the research on this concept is carried out by coupling paradigmatic chaotic systems such as Lorenz and Rössler. Lorenz’s coupling driving Rössler reported an increment in Rössler’s bandwidth [30]. The unidirectional coupling of these two, taking Lorenz as the master and Rössler as the slave, increases the range of parameters, which induces a chaotic solution in Rössler. However, by coupling them bidirectionally, Rössler’s chaotic behavior turns periodic, implying bifurcation’s power in couplings [31]. Another byproduct of couplings is the changes in the phase-space structure of attractors. Based on such observations, coupling schemes capable of retaining the uncoupled attractor have been designed [32].

The subject of the present report is to investigate the interplay of coupled non-identical chaotic oscillators based on the similarity of each strange attractor to its uncoupled state. By increasing coupling strength, the dynamics of the systems are forced to one another, impairing the phase-space structure of attractors. Each attractor is intrinsically prone to different degrees of structural impairment. We aim to quantify the extent of these changes by computing a cost function to find the attractor with lower impairment. Feasible means of comparison are provided by cost ranges, meaning that, in each coupling case, the attractor with a lower cost range is more resilient to structural change. In this paper, we call such an attractor the dominant. Three paradigmatic chaotic systems, Lorenz, Rössler, and Hindmarsh–Rose, are considered. The factors of amplitude and frequency are also examined by scaling, as attractors evolve through different speeds, and they vary in size. Finally, the largest Lyapunov exponent (LLE) of attractors is calculated. It is revealed that the attractor with the larger LLE is less disturbed as the effect of coupling, hence, can be considered as the dominant attractor.

Our results may apply to any conditions where the emergence of weak connections between different oscillators could impair their primary function. As each oscillator’s inherent dynamics is considered, any factor impairing it would be functionally important. For instance, the brain’s connectivity is a prominent feature in the brain based on the neuroanatomy. Circuits consisting of various types of neurons are observed throughout the nervous system [33]. An example of connectivity among a diverse set of neurons is cortico-subcortical basal ganglia circuits that carry out the vital task of movement modulation. In simple terms, if we take the basal ganglia as a controller based on its function (facilitating voluntary and inhibiting unwanted movements [34]), this circuit is a closed loop in which cortical neurons provide the input of the controller, and the desired response is inserted back into the frontal lobe through the thalamus [35]. Any abnormal communication between neural elements may lead to the malfunction of the whole circuit. The term abnormal means stronger or weaker than its functioning state. In the case of Parkinson’s disease, the strengths of former connections are altered, which leads to tremors at rest or a paucity of volunteer movement [36]. Assuming that under pathological conditions, connections in primary pathways are weakened, neighboring neurons exert disturbing inputs to the abovementioned components. Taking the intrinsic dynamics of a neural element in a functioning circuit as primary and its disturbing dynamics as secondary, we can point out some dualities between this scenario and our study. The operative tasks of neighboring neurons can be examined through the coupling of non-identical systems, and low coupling strengths simulate their weak connections. The functional dynamics of oscillators are deemed to be their uncoupled attractors.

In Section 2, as materials of the study, we briefly review the mathematical description, time series, attractor properties of systems, and also the coupling scheme. Then, we introduce the time and amplitude scaling and the comparison methods. In Section 3, we evaluate networks of two non-identical systems and discuss the costs in given coupling strength ranges. The return maps of coupled systems are provided to visualize the relative differences in cost values. By the end of this section, we present the LLEs of systems and their values after scaling. We conclude the results in Section 4.

## 2. Materials and Methods

In this section, the used chaotic systems are described mathematically. To investigate the effect of the amplitude and frequency of oscillations, amplitude and frequency scaling are defined further. The end of this section introduces the applied method for evaluating the similarity of attractors of coupled systems with their original attractors.

### 2.1. Coupled Systems

To investigate the effects of different factors on the dominant attractor of two coupled non-identical oscillators, we consider three paradigmatic chaotic systems: the Lorenz system, the Rössler system, and the Hindmarsh–Rose system. The dynamical equations of these systems are as follows:

Lorenz system:

(1) $$\begin{array}{c} \dot{x}=\sigma \;(y\;-\;x)\\ \dot{y}=x\;(\rho \;-\;z)\;-\;y\\ \dot{z}=\mathrm{xy}\;-\;\beta_{1}z \end{array}$$

where the parameters are σ=10, ρ=28, and $\beta_{1}=2$.

Rössler system:

(2) $$\begin{array}{c} \dot{x}=-\;y\;-\;z\\ \dot{y}=x\;+\;\alpha y\\ \dot{z}=\beta_{2}\;+\;(x\;-\;\gamma )z \end{array}$$

where α=0.2, $\beta_{2}=0.2$, and γ=9.

Hindmarsh–Rose system:

(3) $$\begin{array}{c} \dot{x}{\;=y+3x}^{2\;}{-\;x}^{3\;}-\;z+I\\ \dot{y}{\;=1\;-\;5x}^{2}-\;y\\ \dot{z}\;=-\mathrm{rz}+\mathrm{rs}(x+1.6) \end{array}$$

where I=3.2, r=0.006, and s=4.

Note that the systems at the defined parameters are monostable.

Some of the previous studies [37,38,39] have attempted to investigate the synchronization of coupled chaotic systems and find the best coupling variable for optimal synchronization. As, in this study, the aim is not to reach synchronization, for simplicity, we consider that the systems are coupled linearly through x variables. Considering the describing equations of two systems as ${F=(f}_{1}{,f}_{2}{,f}_{3})$ and ${G=(g}_{1}{,g}_{2}{,g}_{3})$, the coupled systems can be described by

(4) $$\begin{array}{cc} {\dot{x}}_{1}{=f}_{1}\left(x_{1}{,y}_{1}{,z}_{1}\right)+d\left(x_{2\;}{-\;x}_{1}\right)\; & \;{\dot{x}}_{2}{=g}_{1}{(x}_{2}{,y}_{2}{,z}_{2}{)+d(x}_{1\;}{-\;x}_{2})\\ {\dot{y}}_{1}{=f}_{2}\left(x_{1}{,y}_{1}{,z}_{1}\right)\; & \;{\dot{y}}_{2}{=g}_{2}{(x}_{2}{,y}_{2}{,z}_{2})\\ {\dot{z}}_{1}{=f}_{3}\left(x_{1}{,y}_{1}{,z}_{1}\right)\; & \;{\dot{z}}_{2}{=g}_{3}{(x}_{2}{,y}_{2}{,z}_{2}) \end{array}$$

where d denotes the coupling strength. After coupling two systems, the coupling strength is increased, and the attractors of the two coupled systems are compared with their original ones.

**Figure 1:**
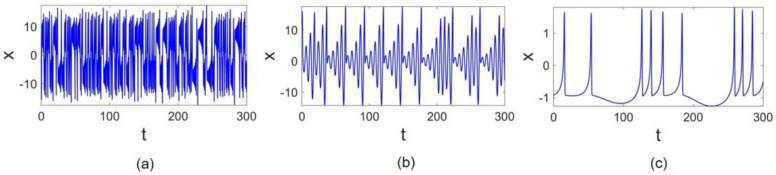
Time series: (**a**) Lorenz x state time series, (**b**) Rössler x state time series, and (**c**) Hindmarsh–Rose x state time series.

The time series of x states of the Lorenz, Rössler, and Hindmarsh–Rose (HR) systems are represented in Figure 1a–c, respectively. It can be observed that the frequency of oscillations is different in these systems. The Lorenz oscillations are faster than the other two, and the HR system is slowest. The time scale of the chaotic oscillations is equal: $f_{L}=0.94$ for the Lorenz system, $f_{R}=0.17$ for the Rössler system, and $f_{HR}=0.03$ for the HR system.

This frequency difference can significantly affect the dynamics of systems after coupling. To examine this effect, we scale the frequencies of systems to have almost the same frequencies. Thus, the parameter ω is defined as the ratio of frequencies between two systems, i.e., the ratio of the frequency of the faster attractor to the slower one. Then, the frequency scaling is performed by taking dt→dt/ω in the equations of the slower system in Equation (4) (the faster system remains unchanged). Consequently, considering the frequency-adjusting parameter, the frequency of the slower system equates with the faster one.

**Figure 2:**
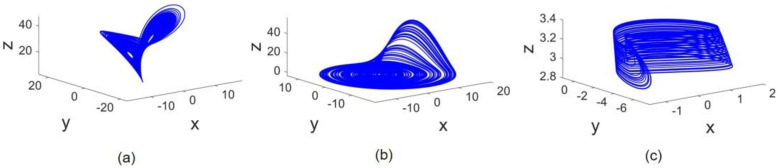
Attractors 3D view: (**a**) Lorenz attractor, (**b**) Rössler attractor, and (**c**) Hindmarsh–Rose attractor.

The attractors of the systems in 3D phase space are depicted in Figure 2. The Lorenz, Rössler, and Hindmarsh–Rose attractors are demonstrated in parts a, b, and c, respectively. As can be seen, the size of the attractors is different. As systems are coupled on x states, the impact of such a dissimilarity only in the x state draws our attention. For example, the difference in Lorenz (or Rössler) attractor size in comparison with Hindmarsh–Rose is noticeable. Therefore, when x states of Lorenz and HR systems are connected, the effect of Lorenz on HR is much higher than vice versa. To examine the impact of the x state amplitudes, a scale on amplitude is also performed.

For scaling the domain of the x state in each system, the absolute global maximum of the x state (${|\mathrm{maxX}}_{A}|$) and the absolute global minimum (${|\mathrm{minX}}_{A}|$) are considered. Then, a scaling parameter a is defined:

(5) $${a=\max\;\{\;|\mathrm{maxX}}_{A}{|,\;|\mathrm{minX}}_{A}\left|\;\right\}$$

By converting x → ax in all equations of the system, the x domain becomes a subset of [−1, 1].

### 2.2. Comparison of the Attractors

A suitable similarity indicator is required to detect the error between the original attractor and the one resulting from adding the coupling term. In the literature, some methods have been proposed for quantifying the difference between attractors [40,41]. Rybin et al. [41] proposed a method to estimate the differences between two return maps based on the 2D histogram. Jafari et al. proposed an objective function for parameter estimation in which the similarity of the attractor’s topologies was employed [40]. Although we do not seek a feasible solution to an optimization problem in this paper, the same approach can provide us with the proper means of similarity estimation, which is our case study. Applying the method proposed by Rybin et al. [41] may also provide improved results, which is suggested to be considered for future works.

**Figure 3:**
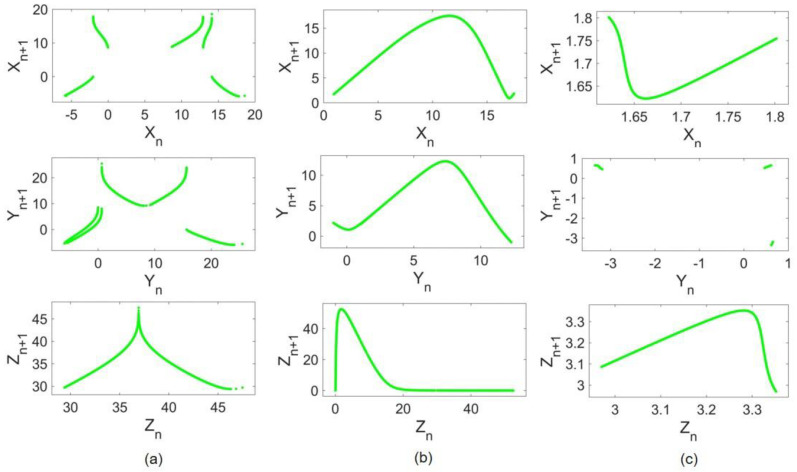
Return maps: (**a**) Lorenz return maps, (**b**) Rössler return maps, and (**c**) Hindmarsh–Rose return maps.

To apply the method, return maps of systems before and after coupling implementation are constructed. A return map (or Poincaré map) is an ordered pattern of a chaotic flow obtained by recording local maxima in a given run time. Local maxima samples constitute a time series P(n). The return map is a plot of P(n+1) as a function of P(n). Figure 3 shows the ordered patterns of return maps obtained from original cases. In Figure 3a, all three return maps of Lorenz states are depicted. Any given initial condition in the basin of attraction of the same attractor results in a similar pattern. Return maps of Rössler and Hindmarsh–Rose are illustrated in Figure 3b,c, respectively.

The proposed cost function is based on calculating the Euclidean distance between original system samples and samples of the same oscillator after the addition of coupling. Considering N samples from the original system and M samples of the coupled oscillator, the algorithm consists of the following steps:

(a) For each point of the original pattern, Euclidean distances with all M points of the coupled oscillator pattern are calculated. The smallest distance among these pairs of points is found. Then, a dataset with size N is obtained, where its summation is $S_{N}$.
(b) For each point of the coupled system pattern, Euclidean distances with all N points of the original pattern are calculated. The smallest distance among these pairs of points is found. Then, a set of data with size M is obtained, and its summation is $S_{M}$.

Then, we take the cost function of each state to be the average of all calculated minimum distances over the whole dataset:

(6) $$\mathrm{Cost}=\frac{S_{M}{+S}_{N}}{M+N}$$

Note that in each step, we have three datasets for each state x, y, and z. Thus, the final value of cost for each system is as follows:

(7) $$\mathrm{Cost}={\mathrm{Cost}}_{x}\;\times \;{\mathrm{Cost}}_{y}\times \;{\mathrm{Cost}}_{z}$$

Of course, a fair number of local maxima from original attractors is required, so we must have enough data in hand to plot return maps. This provides us with maximally covered patterns, and the possible error arising from the paucity of data is minimized. Here, an average of 5000 samples is taken to plot original patterns. The number of local maxima in coupled systems need not be the same as the original data.

## 3. Results

As previously mentioned, the network of two non-identical systems is considered, and the coupling strength varies. The introduced cost function is calculated for each system, and its similarity with the original attractor is found. The fourth-order Runge–Kutta method is used to provide the numerical solutions of differential equations. The required computational time of each coupling case varies according to the speed at which they evolve. These intervals, concerning the integrational steps of 0.005, are long enough to construct return maps with sufficient local maxima samples. They also vary when systems are scaled in frequency. Consequently, the used computational time for each case is mentioned in the corresponding section. Moreover, 40% of data are omitted to avoid any transient. Initial conditions are taken from the original trajectories.

As the coupling strength of the network increases, the interaction of oscillators changes the inherent dynamics of the systems. Therefore, as well as the quantitative changes in each oscillator’s fluctuations, qualitative alternations may occur in the system dynamics, such as bifurcations or unboundedness. Under such conditions, the value and trend of the cost function are no longer valid to determine the overcoming attractor (as the former attractor no longer exists). Given the aim of the study, such alternations in the quality of system dynamics are undesirable. Thus, parameter d is taken within a range in which these conditions are satisfied.

The following results are presented for three different coupling configurations of Lorenz, Rössler, and HR systems. For each coupling configuration, three cases are considered:

(a) Scaling the frequency;
(b) Scaling the x state amplitude;
(c) Applying both (a) and (b) simultaneously.

For each case, the cost functions are computed. The frequency and amplitude scaling results are shown in blue, red, and green colors.

### 3.1. Lorenz–Rössler Coupling

The network of coupled Lorenz and Rössler systems is described in Equation (8).

(8) $$\begin{array}{cc} {\dot{x}}_{L\;}=\sigma \left(y_{L}{\;-\;x}_{L}\right)+d\left(x_{R\;}{-\;x}_{L}\right) & \;{\dot{x}}_{R}{=-\;y}_{R}{-\;z}_{R\;}+d\left(x_{L\;}{-\;x}_{R}\right)\\ {\dot{y}}_{L}{=x}_{L}\left({\rho \;-\;z}_{L}\right){\;-\;y}_{L} & \;{\dot{y}}_{R}{=x}_{R\;}{+\;\alpha y}_{R}\\ {\dot{z}}_{L}{=x}_{L}y_{L}{-\;\beta }_{1}z_{L} & \;{\dot{z}}_{R}{=\beta }_{2}+\;\left(x_{R\;}-\;\gamma \right)z_{R} \end{array}$$

For these systems, the coupling coefficient is raised to d=0.1 as the analyses have shown that no qualitative change occurs in this range. As shown in Figure 1, the Rössler fluctuations are less frequent than the Lorenz system. Therefore, the frequency scaling is applied to make Rössler as fast as Lorenz. Thus, Equation (8) is transformed into Equation (9):

(9) $$\begin{array}{cc} {\dot{x}}_{L}=\sigma \left(y_{L\;}{-\;x}_{L}\right)+d\left(x_{R\;}{-\;x}_{L}\right) & \;{\dot{x}}_{R}{=\omega }_{R}{[-\;y}_{R}{-\;z}_{R\;}+d\left(x_{L\;}{-\;x}_{R}\right)]\\ {\dot{y}}_{L}{=x}_{L}\left({\rho \;-\;z}_{L}\right)\;{-\;y}_{L} & \;{\dot{y}}_{R}{=\omega }_{R}{[x}_{R}\;{+\;\alpha y}_{R}]\\ {\dot{z}}_{L}{=x}_{L}y_{L}\;{-\;\beta }_{1}z_{L} & \;{\dot{z}}_{R}{=\omega }_{R}{[\beta }_{2}\;+\;\left(x_{R\;}-\;\gamma \right)z_{R}] \end{array}$$

where $\omega_{R}=5.53$.

To apply the amplitude scaling, two control parameters are required, which are the absolute value of extrema (maxima or minima) of the x state in each system. Taking the x amplitudes of Lorenz ($C_{L}$) and Rössler ($C_{R}$) as control parameters, Equation (8) turns into Equation (10):

(10) $$\begin{array}{cc} {\dot{x}}_{L}=\frac{1}{C_{L}}[\sigma \left(y_{L\;}{-\;x}_{L}C_{L}\right)+d\left(x_{R}C_{R\;}{-\;x}_{L}C_{L}\right)] & \;{\dot{x}}_{R}=\frac{1}{C_{R}}{[-\;y}_{R}{-\;z}_{R}+d\left(x_{L}C_{L\;}{-\;x}_{R}C_{R}\right)]\\ {\dot{y}}_{L}{=x}_{L}C_{L}\left({\rho \;-\;z}_{L}\right)\;{-\;y}_{L} & \;{\dot{y}}_{R}{=x}_{R}C_{R}\;{+\;\alpha y}_{R}\\ {\dot{z}}_{L}{=x}_{L}C_{L}y_{L\;}{-\;\beta }_{1}z_{L} & \;{\dot{z}}_{R}{=\beta }_{2\;}+\;\left(x_{R}C_{R}\;-\;\gamma \right)z_{R} \end{array}$$

where $C_{L}=17.96$ and $C_{R}=17.49$.

Both frequency and amplitude scaling are performed by combining Equations (9) and (10). To avoid repetition, the resulting equation is not presented. The equations are solved for 7000 time units in general form and for 5000 time units after frequency scaling.

**Figure 4:**
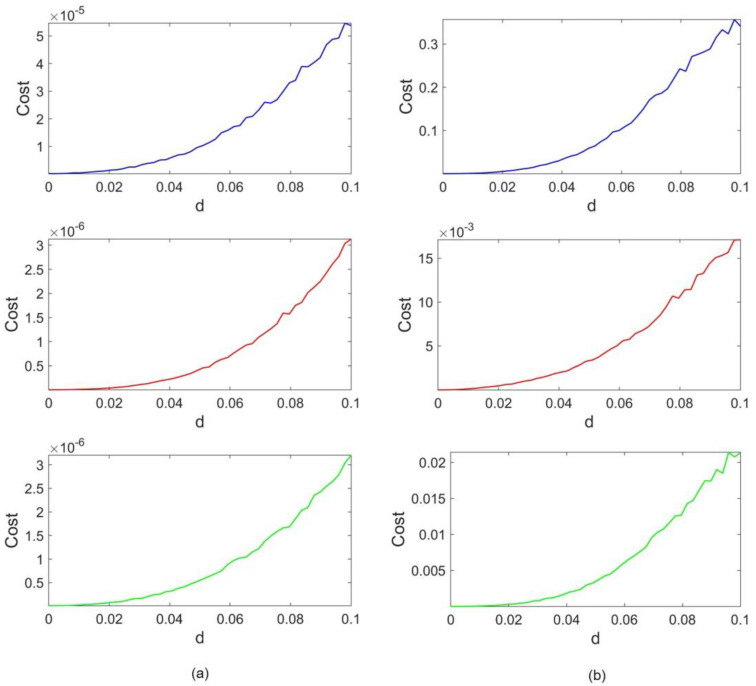
Lorenz–Rössler coupling costs: (**a**) Lorenz costs and (**b**) Rössler costs. The blue, red, and green lines correspond to frequency scaling, x amplitude scaling, and both, respectively.

Figure 4 represents the cost functions concerning the coupling strength. Lorenz and Rössler’s costs are depicted in Figure 4a,b, respectively. The blue, red, and green cost functions refer to the case of frequency scaling, amplitude scaling, and both, respectively. The coupling strength is raised to d = 0.1 to observe the evident growth in cost trends. As it is shown in Figure 4, for d=0 where no coupling is between oscillators, the cost is also equal to zero as each attractor is precisely the same as the original one. By increasing d, a gradual rise appears in the value of the costs. It can be observed that for all three cases, the Rössler cost range is 10,000 times higher than the Lorenz range. To be specific, in the absence of amplitude scaling, the Lorenz cost range is on the order of 10^−5^ and the Rössler cost is within the range of 10^−1^. As amplitudes are scaled, the former ranges convert to 10^−6^ and 10^−2^, respectively. Thus, regardless of the difference in frequency and attractor size, the cost ranges of Lorenz are much smaller than the cost ranges in Rössler. This implies that Rössler’s dynamics do not succeed in affecting Lorenz’s dynamics as much as Lorenz impairs the Rössler attractor. Therefore, it can be concluded that the Lorenz attractor overcomes the Rössler attractor in the given range of coupling strength.

**Figure 5:**
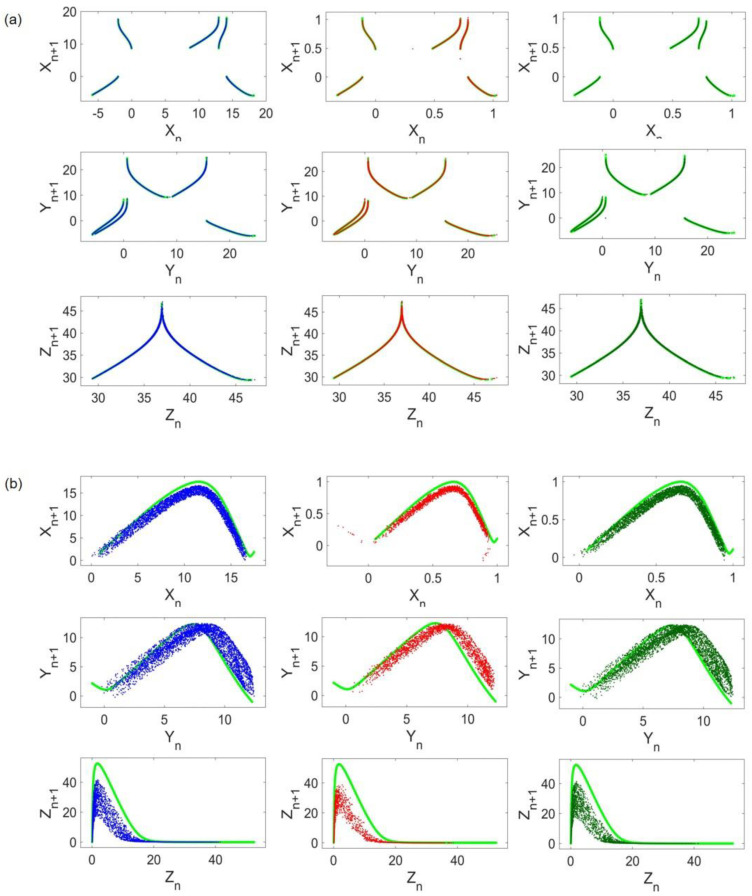
Coupled Lorenz and Rössler return maps: (**a**) Lorenz return maps; (**b**) Rössler return maps. Light green plots represent uncoupled systems return maps. The blue, red, and dark green dots correspond to frequency scaling, x amplitude scaling, and both, respectively.

For visualization purposes, return maps of Lorenz and Rössler systems when the coupling strength is set to d=0.05 are shown in Figure 5a,b, respectively. The left, middle, and right columns refer to the frequency scaling, amplitude scaling, and both. Return maps of uncoupled systems are plotted with light green in the background to compare original and coupled patterns. As shown in Figure 5a, the Lorenz attractor almost remains unchanged in all cases. On the other hand, Rössler samples are less ordered and the coupling results in a different and scattered return map compared with its original pattern (Figure 4b).

### 3.2. Lorenz–Hindmarsh–Rose Coupling

The network of coupled Lorenz and Hindmarsh–Rose systems is described in Equation (11).

(11) $$\begin{array}{cc} {\dot{x}}_{L}=\sigma \left(y_{L\;}{-\;x}_{L}\right)+d\left(x_{H}\;{-\;x}_{L}\right) & \;{\dot{x}}_{H}{=y}_{H}{+3x}_{H}{}^{2}\;{-\;x}_{H}{}^{3}\;{-\;z}_{H\;}+I+d\left(x_{L\;}{-\;x}_{H}\right)\\ {\dot{y}}_{L}{=x}_{L}\left({\rho \;-\;z}_{L}\right)\;{-\;y}_{L} & \;{\dot{y}}_{H}{=1\;-\;5x}_{H}{}^{2}\;{-y}_{H}\\ {\dot{z}}_{L}{=x}_{L}y_{L\;}{-\;\beta }_{1}z_{L} & \;{\dot{z}}_{H}{=-\;\mathrm{rz}}_{H}{+\mathrm{rs}(x}_{H\;}+1.6) \end{array}$$

In this case, the coupling strength varies within the range [0,0.1] in which the attractors are only affected quantitatively. As previously mentioned in Section 2.1, Hindmarsh–Rose evolves much slower than Lorenz. Thus, in this coupling case, the frequency of the HR system is scaled. Under this transformation, Equation (11) changes to Equation (12):

(12) $$\begin{array}{cc} {\dot{x}}_{L}=\sigma \left(y_{L}{\;-\;x}_{L}\right)+d\left(x_{H\;}{-\;x}_{L}\right) & \;{\dot{x}}_{H}{=\omega }_{H}{[\;y}_{H}{+3x}_{H}{}^{2}\;{-\;x}_{H}{}^{3}\;{-\;z}_{H\;}+I+d\left(x_{L\;}{-\;x}_{H}\right)]\\ {\dot{y}}_{L}{=x}_{L}\left({\rho \;-\;z}_{L}\right){\;-\;y}_{L} & \;{\dot{y}}_{H}{=\omega }_{H}{[1\;-\;5x}_{H}{}^{2}\;{-y}_{H}]\\ {\dot{z}}_{L}{=x}_{L}y_{L}{-\;\beta }_{1}z_{L} & \;{\dot{z}}_{H}{=\omega }_{H}{[-\;\mathrm{rz}}_{H}{+\mathrm{rs}(x}_{H\;}+1.6)] \end{array}$$

where $\omega_{H}=31.25$.

To study the role of size of attractors in the results, x state amplitudes are scaled by two control parameters $C_{L}$ and $C_{H}$ that are Lorenz and HR scaling parameters, respectively. Thus, Equation (11) is transformed into Equation (13):

(13) $$\begin{array}{cc} {\dot{x}}_{L}=\frac{1}{C_{L}}[\sigma \left(y_{L\;}{-\;C}_{L}x_{L}\right)+d\left(C_{H}x_{H\;}{-\;C}_{L}x_{L}\right)] & \;{\dot{x}}_{H}=\frac{1}{C_{L}} [y_{H}+3{\left(C_{H}x_{H}\right)}^{2\;}{-\;(C}_{H}x_{H}{)}^{3}\;{-\;z}_{H\;}+d\left(C_{L}x_{L}\;-\;C_{H}x_{H}\right)]\\ {\dot{y}}_{L}{=C}_{L}x_{L}\left({\rho \;-\;z}_{L}\right)\;{-\;C}_{L}y_{L} & \;{\dot{y}}_{H}{=1\;-\;5(C}_{H}x_{H}{)}^{2}\;{-\;y}_{H}\\ {\dot{z}}_{L}{=C}_{L}x_{L}y_{L\;}{-\;\beta }_{1}z_{L} & \;{\dot{z}}_{H}{=-\;\mathrm{rz}}_{H\;}{+\mathrm{rs}(C}_{H}x_{H}+1.6) \end{array}$$

where $C_{L}=17.96$ and $C_{H}=$1.8.

To achieve the equations of the Lorenz–HR network after both frequency and amplitude scaling, it suffices to combine Equations (12) and (13). To avoid repetition, the resulting equation is not represented. Due to the slow dynamics of HR, simulations are performed up to 50,000 time units. When the frequency of HR is scaled, a computational time of 7000 suffices.

**Figure 6:**
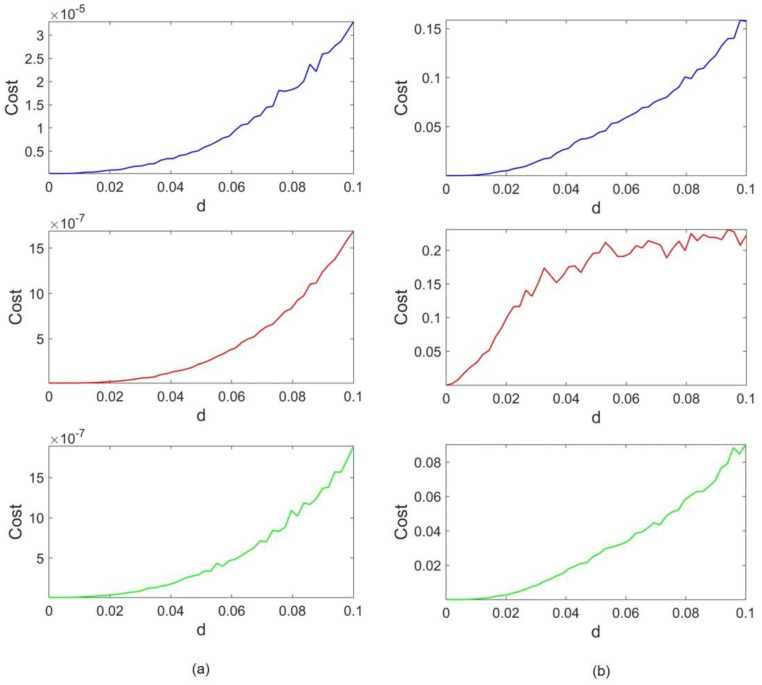
Lorenz–HR coupling costs: (**a**) Lorenz costs; (**b**) HR costs. The blue, red, and green lines correspond to frequency scaling, x amplitude scaling, and both, respectively.

The cost functions concerning coupling coefficient d are provided in Figure 6. Lorenz and HR costs are represented in Figure 6a,b, respectively. Similar to the previous case, the coupling strength is elevated until d = 0.1. We observe that the value of costs when no coupling is applied (d=0) is zero, and an incremental trend is seen as coupling strength increases. Furthermore, in all cases, the Lorenz cost range is much smaller than the HR cost range, implying that despite the type of applied scaling, the overcoming attractor is Lorenz. The HR cost ranges tend to be 10,000 higher than those of Lorenz. When both scalings are applied, the HR cost slightly improves and lowers from 0.1575 and 0.22 down to 0.09. On the other hand, the Lorenz cost is also enhanced, and the ratio of HR and Lorenz costs remains the same.

The trend of HR cost in the case of x amplitude scaling (red plot) differs considerably from the HR cost in two other cases. This dissimilarity is well recognizable at d=0.01 as cost values in blue and green graphs (when frequency scaling is applied, whether x is scaled or not) are approximately equal to zero. In contrast, the cost does not equate to zero in the red plot (where HR frequency is not scaled). Although the dominant attractor is the Lorenz attractor, it can be deemed that slow HR is more prone to be impaired by Lorenz.

**Figure 7:**
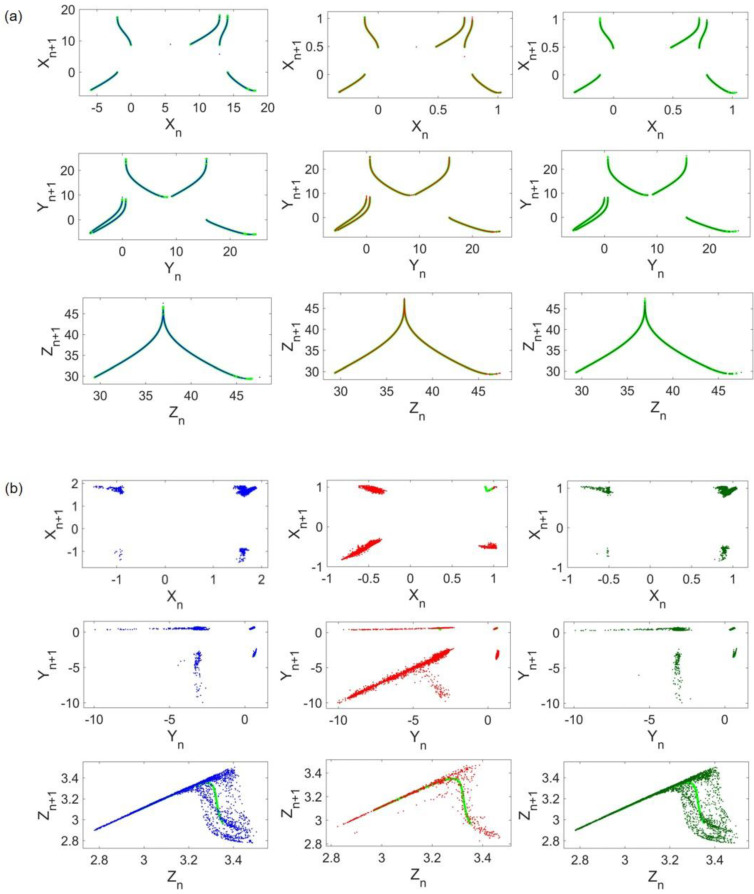
Coupled Lorenz and Hindmarsh–Rose return maps: (**a**) Lorenz return maps; (**b**) Hindmarsh–Rose return maps. Light green plots represent uncoupled systems return maps. The blue, red, and dark green dots correspond to frequency scaling, x amplitude scaling, and both, respectively.

To investigate the abovementioned difference in HR costs, return maps of Lorenz and HR systems, when the coupling strength is d=0.01, are provided in Figure 7. For comparison, original patterns are also shown by light green in the background. Visual evidence in Figure 7a confirms the low value of Lorenz cost as the coupled Lorenz has its former return map patterns in all cases. HR return maps are illustrated in Figure 7b, indicating that coupled HR return map patterns, notably in x and y states, are thoroughly impaired by the emergence of scattered points. In general, distributed points of coupled HR seem to construct a rough order. However, this order differs when no frequency scaling is performed. To be more accurate, in the red graph, a different line of points that is nowhere near the original pattern appears, and fewer points exist near the original pattern of the HR x state. This reasoning seems to justify the previously mentioned cost difference.

Unlike the results obtained from Lorenz–Rössler coupling, Lorenz–HR coupling indicates that, when non-identical oscillators vary notably in the time scale, matching frequencies might somewhat help the slower system (in this case, HR) to preserve its attractor. Note that the frequency-adjusting parameter of HR in Lorenz–HR coupling is $\omega_{H}=31.25$, whereas in Lorenz–Rössler coupling, it is $\omega_{R}=5.42$.

### 3.3. Rössler–Hindmarsh–Rose Coupling

The equations of our last case of study, the coupling of Rössler and Hindmarsh–Rose, are as follows:

(14) $$\begin{array}{cc} {\dot{x}}_{R}{=-\;y}_{R}\;{-\;z}_{R\;}+d\left(x_{H\;}{-\;x}_{R}\right) & \;{\dot{x}}_{H}{=y}_{H}{+3x}_{H}{}^{2}\;{-\;x}_{H}{}^{3}\;{-\;z}_{H}+I+d\left(x_{R\;}{-\;x}_{H}\right)\\ {\dot{y}}_{R}{=x}_{R\;}{+\;\alpha y}_{R} & \;{\dot{y}}_{H}{=1\;-\;5x}_{H}{}^{2}\;{-\;y}_{H}\\ {\dot{z}}_{R}{=\beta }_{2\;}+\;\left(x_{R\;}-\;\gamma \right)z_{R} & \;{\dot{z}}_{H}{=-\;\mathrm{rz}}_{H\;}{+\mathrm{rs}(x}_{H}+1.6) \end{array}$$

In this case, the slower oscillator is HR. Thus, frequency scaling is applied to HR equations, and the frequency-adjusting parameter is taken as $\omega_{\mathrm{HR}}$ to avoid repetition. With this transformation, Equation (14) changes to Equation (15):

(15) $$\begin{array}{cc} {\dot{x}}_{R}{=-\;y}_{R}\;{-\;z}_{R\;}+d\left(x_{H\;}{-\;x}_{R}\right) & \;{\dot{x}}_{H}{=\omega }_{\mathrm{HR}\;}{[y}_{H}{+3x}_{H}{}^{2}\;{-\;x}_{H}{}^{3}\;{-\;z}_{H}+I+d\left(x_{R\;}{-\;x}_{H}\right)]\\ {\dot{y}}_{R}{=x}_{R\;}{+\;\alpha y}_{R} & \;{\dot{y}}_{H}{=\omega }_{\mathrm{HR}\;}{[1\;-\;5x}_{H}{}^{2}\;{-\;y}_{H}]\\ {\dot{z}}_{R}{=\beta }_{2\;}+\;\left(x_{R\;}-\;\gamma \right)z_{R} & \;{\dot{z}}_{H}{=\omega }_{\mathrm{HR}\;}[{-\;\mathrm{rz}}_{H\;}{+\mathrm{rs}(x}_{H}+1.6)] \end{array}$$

where $\omega_{\mathrm{HR}}=5.76.$

To scale x amplitudes, scaling parameters $C_{R}$ and $C_{H}$ turn Equation (14) to Equation (16):

(16) $$\begin{array}{cc} {\dot{x}}_{R}=\frac{1}{C_{R}}{[\;-\;y}_{R\;}{-\;z}_{R}+d\left(x_{H}C_{H\;}{-\;x}_{R}C_{R}\right)] & \;{\dot{x}}_{H}=\frac{1}{C_{H}} [y_{H}+3{\left(C_{H}x_{H}\right)}^{2}\;{-\;(C}_{H}x_{H}{)}^{3}\;{-\;z}_{H\;}+I+d\left(x_{R}C_{R}{-\;C}_{H}x_{H}\right)]\\ {\dot{y}}_{R}{=x}_{R}C_{R\;}{+\;\alpha y}_{R} & \;{\dot{y}}_{H}{=1\;-\;5(C}_{H}x_{H}{)}^{2}\;{-\;y}_{H}\\ {\dot{z}}_{R}{=\beta }_{2}\;+\;\left(x_{R}C_{R\;}-\;\gamma \right)z_{R} & \;{\dot{z}}_{H}{=-\;\mathrm{rz}}_{H}{+\mathrm{rs}(x}_{H}+1.6) \end{array}$$

where x scaling parameters are set to $C_{R}=17.49$ and $C_{H}=1.8.$

Moreover, to evaluate the roles of time and amplitude scale simultaneously, Equations (15) and (16) are combined. As both coupled systems are slow, a computational time of more than 60,000 is needed. Scaling HR frequency decreases the simulation time to 50,000.

**Figure 8:**
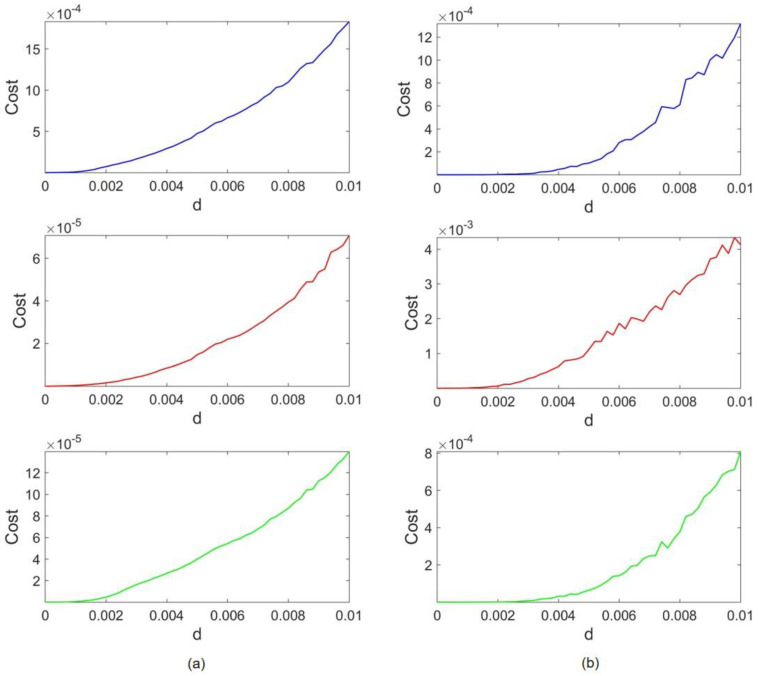
Rössler–HR coupling costs: (**a**) Rössler costs; (**b**) HR costs. The blue, red, and green lines correspond to frequency scaling, x amplitude scaling, and both, respectively.

The cost results of this coupling configuration are depicted in Figure 8. In this case, it suffices to raise the coupling strength to d=0.01, as both attractors become vulnerable as the coupling becomes stronger. Figure 8a,b stand for Rössler and HR costs, respectively. Again, the trends of costs follow the expected pattern, and they grow by increasing the coupling strength. According to the blue plot, Rössler and HR cost orders are within the same range when the HR frequency is scaled to be as fast as Rössler. Although the HR cost is 0.0013 and Rössler owns a cost of 0.0018, this slight difference can be ignored when return maps are analyzed further (both are on the order of $10^{-4}$). However, the Rössler attractor tends to overcome HR when x amplitudes are also scaled (as its cost becomes 10 to 100 times lower than HR cost). Compared to Lorenz–Rössler and Lorenz–HR couplings, cost orders are much closer in this case.

**Figure 9:**
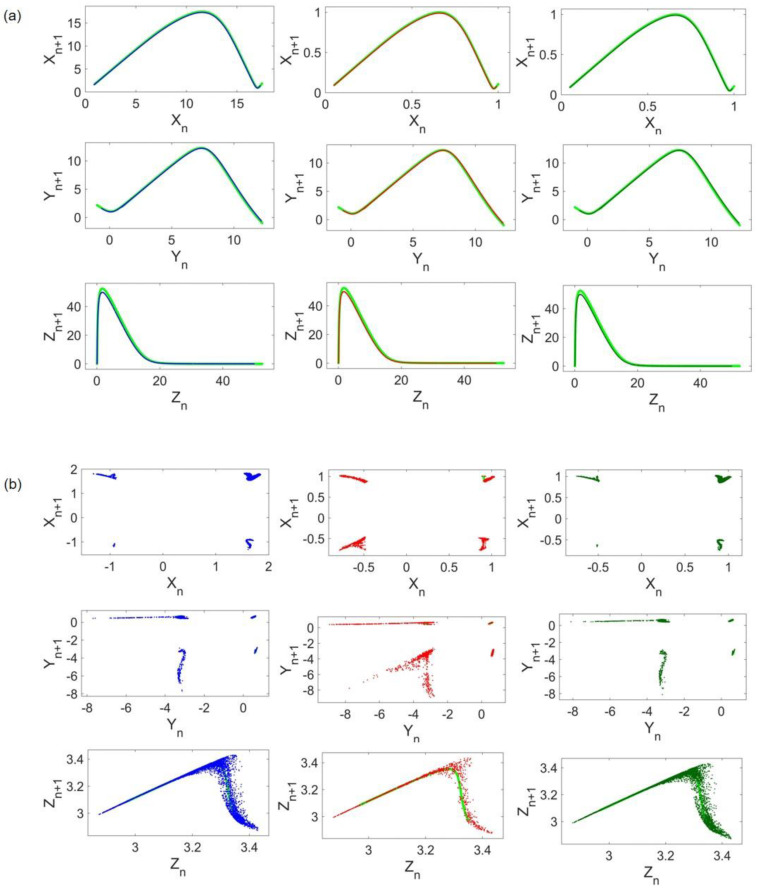
Coupled Rössler and Hindmarsh–Rose return maps: (**a**) Lorenz return maps; (**b**) Hindmarsh-Rose return maps. Light green plots represent uncoupled systems return maps. The blue, red, and dark green dots correspond to frequency scaling, x amplitude scaling, and both, respectively.

To shed light on the dominant attractor, return maps concerning d=0.005 are provided in Figure 9. Comparison of Figure 9a,b, which are the corresponding return maps of Rössler and HR, respectively, implies that the Rössler attractor remains unaltered. Even though Figure 9b shows that adjacent points of the coupled HR return map exist near the original plot (light green in the background), which is the root of its low cost, its pattern changes. Hence, in this case, the Rössler attractor is the dominant attractor.

### 3.4. Lyapunov Exponents of Systems

To compare the inherent characteristics of our studied systems, the largest Lyapunov exponents (LLE) are computed in all cases and presented in Table 1. As can be seen, the most significant value of LLE belongs to the Lorenz system. An important observation is that when the size of an attractor is scaled, it has no impact on LE values. Thus, LLE values of amplitude-scaled systems are not presented, to avoid repetition. However, time scaling affects the LEs of a system directly. For instance, consider the Lorenz–Rössler coupling configuration. When the control parameter scales the Rössler frequency ($\omega_{R}$), its LLE is also multiplied by $\omega_{R}$ (turning 0.08 to 0.433). As the results in previous sections suggest, in Lorenz–Rössler and Lorenz–HR cases, the Lorenz attractor is dominant. In both cases, even by scaling Rössler and HR timing, the Lorenz LLE is twice the LLEs of Rössler and HR. As the cost data in Figure 8 suggest, when the HR frequency is scaled in Rössler–HR coupling, Rössler barely overcomes HR. There is speculation that the reason for such a very close competition lies in the fact that the LLEs of the two attractors are so close (Rössler LLE=0.08 and time-scaled HR LLE=0.075). Consequently, one can generalize that in all cases, the attractor with a greater value of LLE is the dominant one.

Kaplan–York dimensions ($D_{\mathrm{KY}})$ of attractors are also calculated using Lyapunov exponents and are shown in Table 1. Any type of scaling, whether amplitude or frequency, fails to alter the $D_{\mathrm{KY}}$ of attractors.

## 4. Conclusions

This study investigated the dominant attractor in Lorenz–Rössler, Lorenz–HR, and Rössler–HR coupled systems. The similarity of each system’s former and latter attractor was assessed by calculating a cost function based on Euclidean distances of return map samples. The impact of attractor size and system oscillation frequency was also considered. Relative comparisons were carried out between cost ranges in a given period of coupling strength. In the Lorenz–Rössler case, the Lorenz cost range was 10,000 times lower than that of Rössler regardless of scaling in frequency and amplitude. The ratio of costs in Lorenz–HR coupling resulted in the same number of 10,000. In the coupling case of Rössler–HR, costs tended to be within the same range, and the most significant disparities in ratios were within the range of 10 to 100. Altogether, we surmised that an attractor with a lower cost range was the one that succeeds in maintaining its previous dynamics. An illustration of return maps confirmed our speculation based on cost results. It can be concluded that the rerun map of an attractor with a lower cost range tends to preserve its previous patterns. It was obtained that in each case, the attractor with a higher value of LLE dominated the other. Lorenz, with the greatest LLE value of 0.819, held the lowest cost values and the least impairments in the return map pattern. Our observations also implied that in Hindmarsh–Rose coupling cases, scattered points appeared and impaired its return map pattern. The effect of frequency difference was highlighted by the fact that when the HR frequency was scaled, some scattered points were omitted, and its cost was enhanced. The LLE value of HR also supports this outcome as a slow system. When frequency scaling was applied to HR, its LLE was multiplied by the scaling parameter. It raised from 0.013 to 0.075, while Rössler held an LLE value of 0.08. In this case, the closeness in LLE was in line with the close range of Rössler and HR costs. Thus, the raised value of LLE helped HR preserve its attractor. Finally, our results suggest that an attractor size has no role in determining the overcoming attractor, and it is supported by the fact that it does not change LLE values either.

## Figures and Tables

**Table 1:** Largest Lyapunov exponents of systems.

System	LLE	LLE When Frequency Is Scaled with $\omega_{R}=5.42$ (Lorenz–Rössler)	LLE When Frequency Is Scaled with $\omega_{H}=31.25$ (Lorenz–HR)	LLE When Frequency Is Scaled with $\omega_{\mathrm{HR}}=5.76$ (Rössler–HR)	$D_{\mathrm{KY}}$
Lorenz	0.819	0.819	0.819	-	2.059
Rössler	0.08	0.433	-	0.08	2.009
HR	0.013	-	0.406	0.075	2.001

## Data Availability

Not applicable.
